# Body mass index and the outcome of acute respiratory distress syndrome, what is their relationship and why?

**DOI:** 10.1111/crj.13583

**Published:** 2023-01-11

**Authors:** Jin‐Ke Sun, Qing‐Hua Xu

**Affiliations:** ^1^ Department of Pulmonary and Critical Care Medicine Liaocheng People's Hospital Affiliated to Graduate School of Shandong First Medical University Liaocheng China; ^2^ Department of Health Care Liaocheng People's Hospital Liaocheng China

Dear Editor,

We have read with great interest the article entitled ‘The Association between Outcomes and Body Mass Index in Patients with Acute Respiratory Distress Syndrome’ published in the Clinical Respiratory Journal.^1^ The author showed that with the increase of body mass index (BMI), the mortality of acute respiratory distress syndrome (ARDS) dropped gradually. We congratulate the authors and would like to give some points.

In this study, patients were divided into the following four groups: underweight, normal weight, overweight and obese. Although pancreatitis was not a major cause of ARDS, we noticed that the number of pancreatitis of overweight and obese groups was obviously more than that of the first two groups. ARDS could be divided into pulmonary ARDS (ARDSp, such as pneumonia) and extra pulmonary ARDS (ARDSexp, such as pancreatitis) according to risk factors commonly. At present, research data onto mortality comparison between ARDSp and ARDSexp are limited and inconclusive. Study by Shu et al. showed that ARDSp is associated with slower improvement, more noninvasive ventilation (NIV) failure and higher 28‐day mortality than ARDSexp among patients with ARDS who used NIV as a first‐line therapy.^2^ And others believed that treatment based on respiratory mechanics is more effective to improve alveolar recruitment and gas‐exchange in ARDSexp.^3^ We could guess that pancreatitis may be one of the reasons for the low mortality of ARDS patients with high BMI. Unfortunately, the effect of different causes, especially pancreatitis, on mortality of patients with ARDS was not involved in the article.

After reviewing a large number of clinical studies, we found that some researchers supported the author's point of view, but they can hardly cover the extreme BMI.^4^, ^5^ Others considered that the mortality in patients with ARDS was not affected by BMI.^6^, ^7^ Interestingly, Liu et al. suggest that the relationship of mortality with BMI might follow an U‐shaped curve, with increasing mortality in very low BMI and in severe obesity.^8^ In general, although opinions differ, it seems that the outcome of obese patients with ARDS is similar or better than that of non‐obese patients. However, the specific reasons have not been explained clearly.

Now, we would like to consider some possible reasons from a clinical perspective: Firstly, on the premise of similar lung injury, we believe that obese patients may be more prone to hypoxemia than normal weight patients and the probability of bilateral infiltrating atelectasis increases. The latter relatively leads to low oxygenation index (PaO_2_/FiO_2_), which is also easy to be misdiagnosed as ARDS. There is no doubt that these patients have a better outcome. Secondly, clinicians may think that obese patients have a higher risk of poor outcome. This view may lead to early admission of patients to the ICU with increased monitoring and preventive measures. Thirdly, the prevalence of pancreatitis is higher in obese patients, which may reduce the mortality of these patients with ARDS. But this point still needs to be verified by more researches.

Therefore, we think that the outcome of obese patients is affected by some external subjective factors and the view of low mortality in obese patients with ARDS should be revisited. In the future, clinicians should aim to improve the accuracy of ARDS diagnosis. There is a need for large sample studies designed with identically defined treatment protocols, and with same risk factors of ARDS, to further determine the precise roles of BMI in patients with ARDS.

## CONFLICT OF INTEREST

The authors report no relationships that could be construed as a conflict of interest.

## AUTHOR CONTRIBUTIONS

Jin‐Ke Sun design and write this article. Qing‐Hua Xu guide and critically review the main content of the article.

## ETHICS STATEMENT

This study does not involve human and animal experiments.

## Data Availability

Data sharing is not applicable to this article as no new data were created or analysed in this study.
